# Supplementary material for: Controllable photomechanical bending of metal-organic rotaxane crystals facilitated by regioselective confined-space photodimerization
Source: Nat Commun. 2022 Apr 19;13:2030. doi: 10.1038/s41467-022-29738-y (PMC9019062; doi:10.1038/s41467-022-29738-y)
Supplement: Supplementary file 1 — Supplementary Information [file 41467_2022_29738_MOESM1_ESM.pdf]

Supplementary Information

Controllable Photomechanical Bending of Metal-organic Rotaxane Crystals Facilitated by Regioselective Confined-Space Photodimerization

Jun-shan Geng, Lei Mei,* Yuan-yuan Liang, Li-yong Yuan, Ji-pan Yu, Kong-qiu Hu, Li-hua Yuan, Wen Feng,* Zhi-fang Chai, Wei-qun Shi*

Table of Contents

Supplementary Methods

Synthesis procedure of [HMPyVB]I, CB[8]-HMPyVB and photo-dimerized CB[8]-HMPyVB.

Supplementary Figures

Supplementary Fig. 1 ^{1}H NMR spectrum of [HMPyVB]I.
Supplementary Fig. 2 Simulated and experimental PXRD patterns of U-CB[8]-MPyVB.
Supplementary Fig. 3 TGA results of U-CB[8]-MPyVB and U-CB[8]-MPyVB-A.
Supplementary Fig. 4 FT-IR spectra of U-CB[8]-MPyVB and U-CB[8]-MPyVB-A.
Supplementary Fig. 5 Solid state fluorescence spectra of CB[8]-HMPyVB and U-CB[8]-MPyVB.
Supplementary Fig. 6 Single crystal structures of uranyl complexes with pyridium- or viologen-functionalized organic carboxylate linkers.
Supplementary Fig. 7 ^{1}H NMR spectra of HMPyVB$^+$ and CB[8] with different stoichiometric ratios.
Supplementary Fig. 8 The ESI-MS spectrum of $(HMPyVB^+)_2$@CB[8] motif.
Supplementary Fig. 9 Single crystal structure of CB[8]-HMPyVB.
Supplementary Fig. 10 The photoresponsive behavior of U-CB[8]-MPyVB under different UV irradiation time.
Supplementary Fig. 11 Thermal effects on the observed motion of the crystal under UV lamp.
Supplementary Fig. 12 ^{1}H NMR spectrum of model complex $(HMPyVB^+)_2$@CB[8] before and after UV radiation.
Supplementary Fig. 13 Fluorescence spectra of model complex $(HMPyVB^+)_2$@CB[8] before and after UV radiation.
Supplementary Fig. 14 FT-IR spectrum of model complex $(HMPyVB^+)_2$@CB[8] before and after UV radiation.
Supplementary Fig. 15 Single crystal structure of U-CB[8]-MPyVB-A.
Supplementary Fig. 16 Single crystal structure of U-CB[8]-MPyVB-Int.
Supplementary Fig. 17 Single crystal structure of [HMPyVB]I.
Supplementary Fig. 18 ^{1}H NMR spectra of crystalline [HMPyVB]I before and after UV radiation.
Supplementary Fig. 19 ^{1}H NMR spectra of crystalline CB[8]-HMPyVB before and after UV radiation.
Supplementary Fig. 20 HMPyVB$^+$ or MPyVB motifs with different conformations in different environments.
Supplementary Fig. 21 The changes of photoinert motif and photoactive motif before and after UV irradiation.

Supplementary Tables

Supplementary Table 1 Distances between C=C bonds and dihedral angles between two benzene rings of HMPyVB$^+$ or MPyVB ligands in diffirent enviroments.
Supplementary Table 2 Distances between O...O in front and back views of CB[8] with different conformations.
Supplementary Table 3 Crystal and refinement data of [HMPyVB]I**,** CB[8]-HMPyVB**,** U-CB[8]-MPyVB, U-CB[8]-MPyVB-Int and U-CB[8]-MPyVB-A.
Supplementary Table 4 Comparison of cell parameters and response time between U-CB[8]-MPyVB and other photobending materials before and after photodimerization.

Supplementary Methods

Synthesis of [HMPyVB]I:

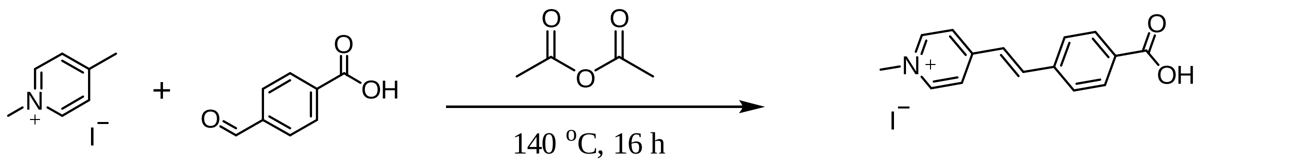

Compound **1** was synthesized according to the previously reported literature without any modification[1]. 1,4-dimethylpyridin-1-ium (12.69 g, 0.054 mol) iodide and 4-formylbenzoic acid (8.00 g, 0.053 mol) were refluxed in 25 mL acetic anhydride for 16 hours. The mixture was then cooled to ambient temperature and poured onto 200 mL of ice water. A light-yellow powder was collected from filtration, and washed repeatedly with water, followed by ethanol aqueous, and dried to give (E)-4-[2-(methylpyridin-4-yl) vinyl]benzoic acid ([HMPyVB]I) (13.9 g, 71.9%). ^{1}H NMR (500 MHz, DMSO-d6): δ/ppm =13.14 (br, 1H), 8.91 (d, 2H), 8.27 (d, 2H), 8.07 (d, 1H), 8.03 (d, 2H), 7.85 (d, 2H), 7.67 (d, 1H), 4.21(s, 3H), Figure S1.

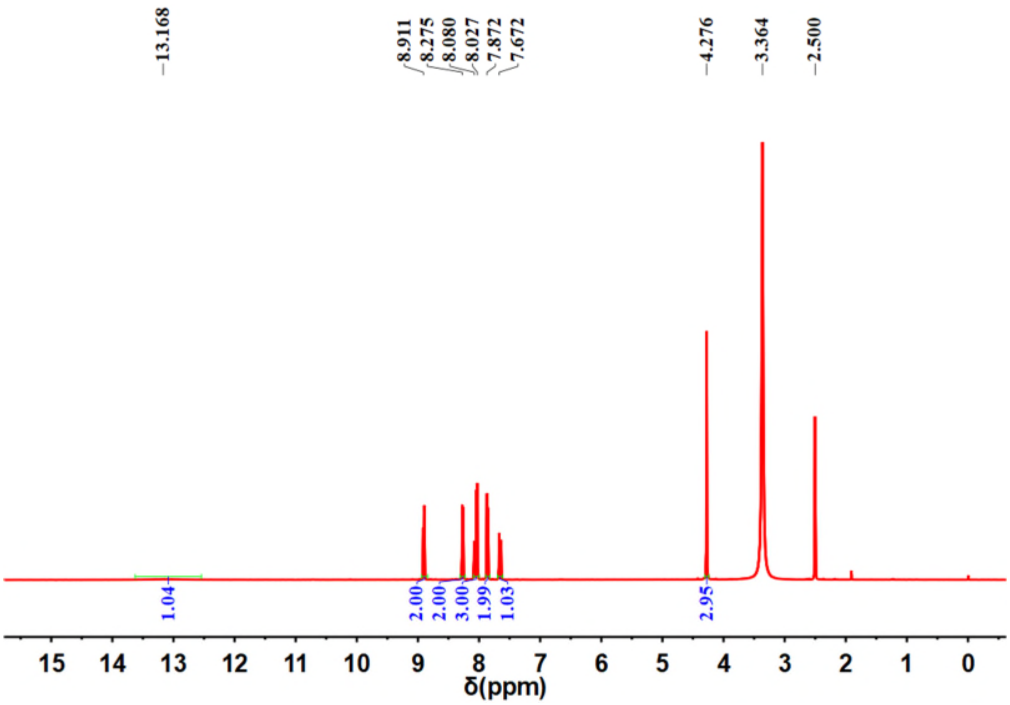

Supplementary Fig. 1 ^{1}H NMR spectrum of [HMPyVB]I at 25 °C (DMSO-d_6, 500 M Hz).

Synthesis of CB[8]-HMPyVB: [HMPyVB]I (18.3 mg, 0.05 mmol) and CB[8] (33.2 mg, 0.025 mmol) were added into a 15 mL polytetrafluoroethylene hydrothermal reactor, and then 2 mL deionized water was added. The solvent was evenly distributed by ultrasonic vibration for 5 minutes. After heating at 150 ℃ for 24 hours and natural cooling to room temperature, light yellow octahedral single crystals was obtained, which was suitable for single crystal X-ray diffraction. The crystals were collected by centrifugation, then washed with deionized water (10 mL) for three times, filtered and dried in vacuum. Finally, 38.4 mg light yellow single crystals were obtained with the yield of 74.6%.

Synthesis of photo-dimerized CB[8]-HMPyVB: In the presence of CB[8], the photodimerization reaction of [HMPyVB]I in solution can be catalyzed to obtain pure photodimerization product encapsulated in CB[8]. Therefore, CB[8] (0.04 mM) was added into the aqueous solution of [HMPyVB]I with the concentration of 0.08 mM. After ultrasonic treatment for 5 minutes, the supramolecular complex $(HMPyVB^+)_2$@CB[8] was formed. The solution was then irradiated for 20 min and evaporated in vacuum to afford light yellow powder of dimerization product of CB[8]-HMPyVB, which was subject to subsequent characterization.

Supplementary Figures

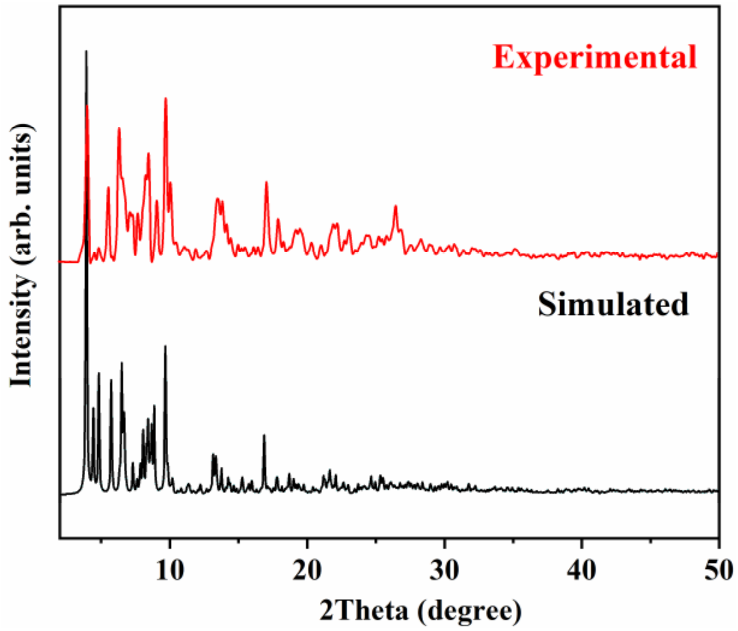

Supplementary Fig. 2 Simulated and experimental PXRD patterns of U-CB[8]-MPyVB.

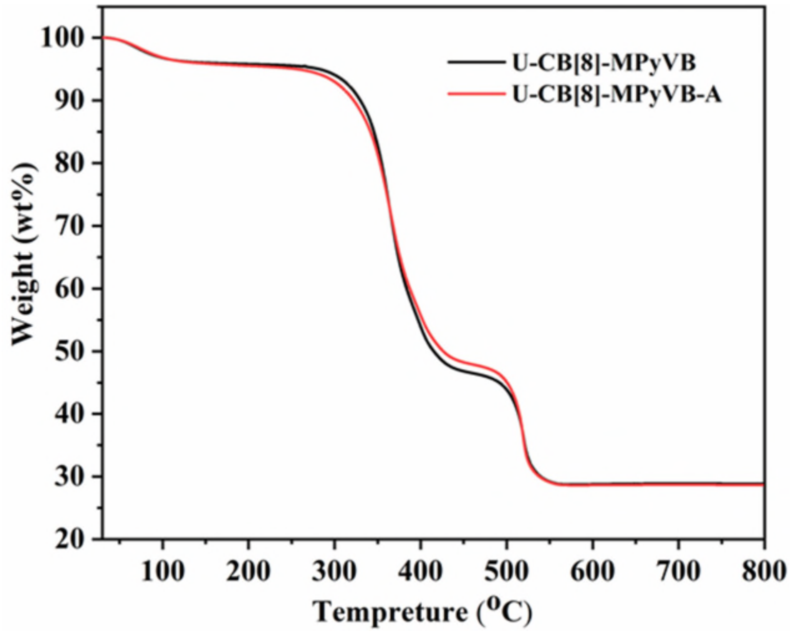

Supplementary Fig. 3 TGA results of U-CB[8]-MPyVB and U-CB[8]-MPyVB-A.

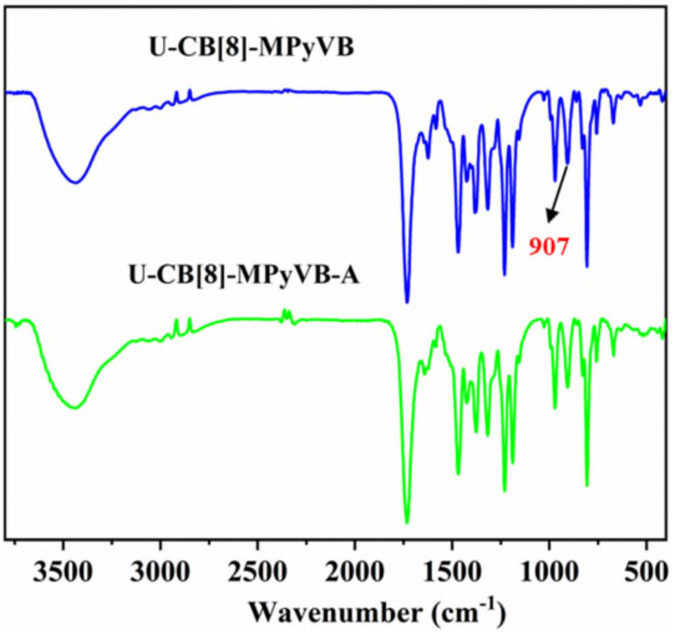

Supplementary Fig. 4 FT-IR spectra of U-CB[8]-MPyVB and U-CB[8]-MPyVB-A (The arrow indicates a typical U=O vibration band at 907 cm^{-1}).

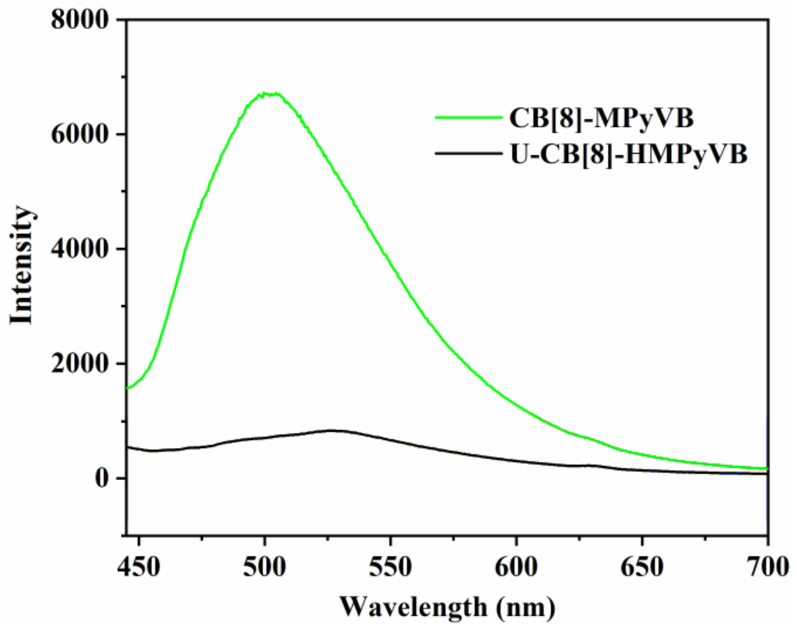

Supplementary Fig. 5 Solid state fluorescence spectra of CB[8]-HMPyVB and U-CB[8]-MPyVB. The excitation wavelength is 420 nm.

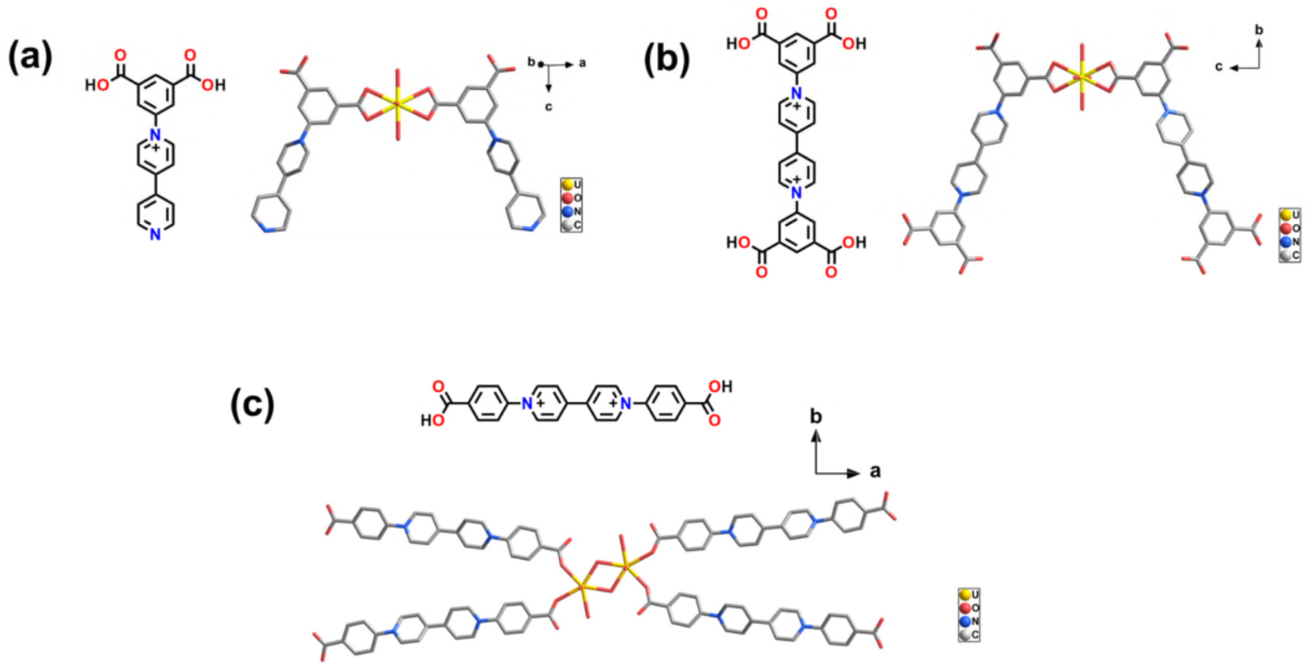

Supplementary Fig. 6 Single crystal structures of uranyl complexes with pyridium- or viologen-functionalized organic carboxylate linkers. **(a)** 1-(3,5-dicarboxyphenyl)-4,4′-bipyridinium chloride (H_2ibpbCl) ligand. **(b)** 1, 1′-bis(3,5-dicarboxyphenyl)-4,4′-bipyridinium chloride ([H_4bdbp·]Cl_2) ligand. **(c)** 1,1′-bis(4-carboxyphenyl)-4,4′-bipyridinium bis chloride ([H_2bcbp]Cl_2) ligand.

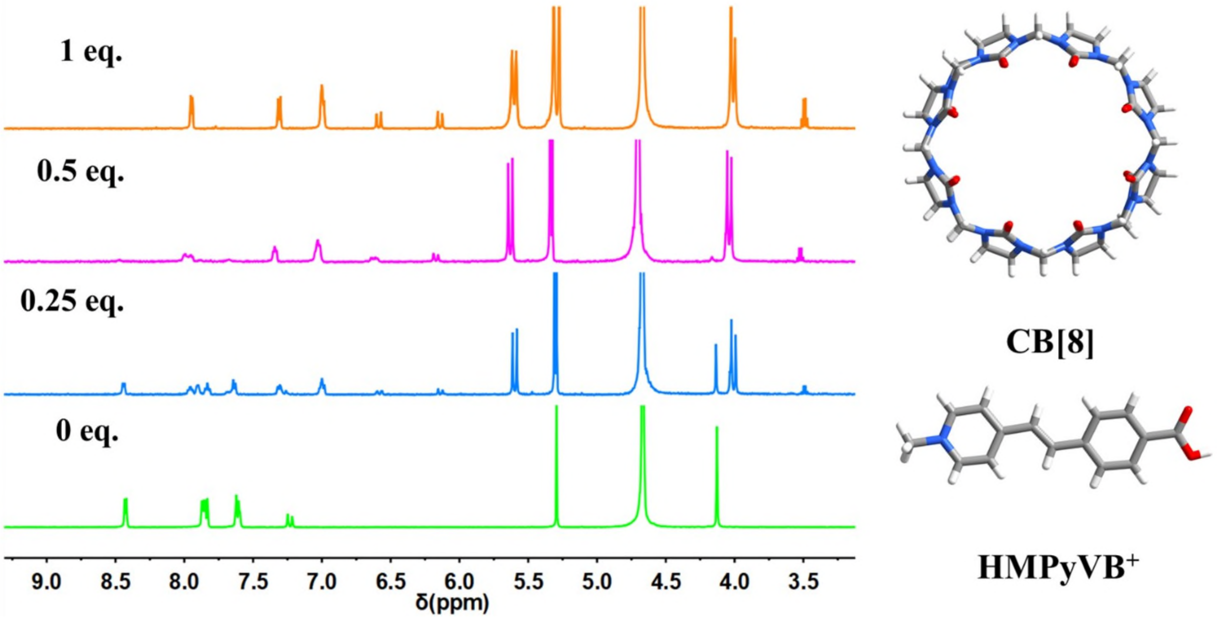

Supplementary Fig. 7 ^{1}H NMR spectra of $HMPyVB^+$ and CB[8] with different stoichiometric ratios at 25 °C (D_2O, 500 M Hz).

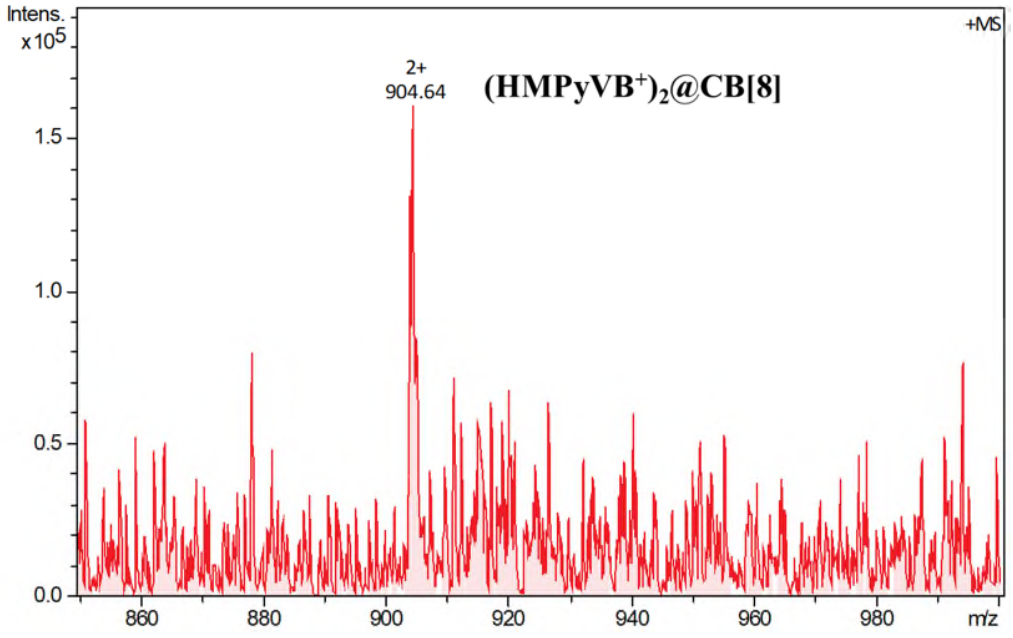

Supplementary Fig. 8 The ESI-MS spectrum of $(HMPyVB^{+})_2$@CB[8] motif.

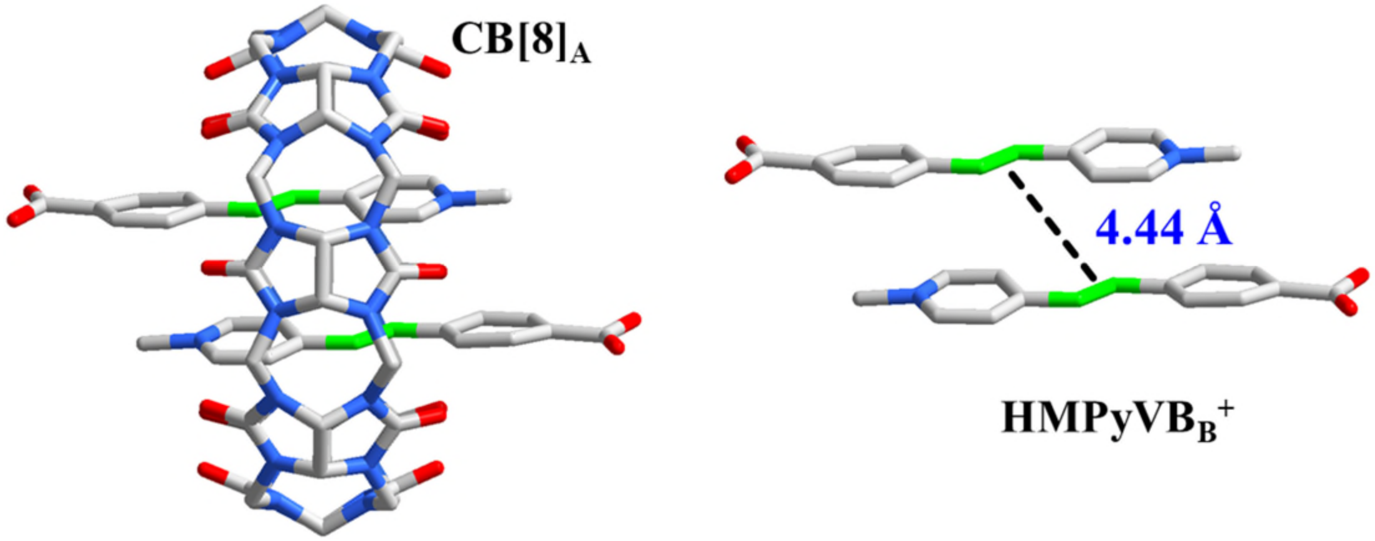

Supplementary Fig. 9 Single crystal structure of CB[8]-HMPyVB and the distance between C=C double bonds of $[HMPyVB]^{+}$.

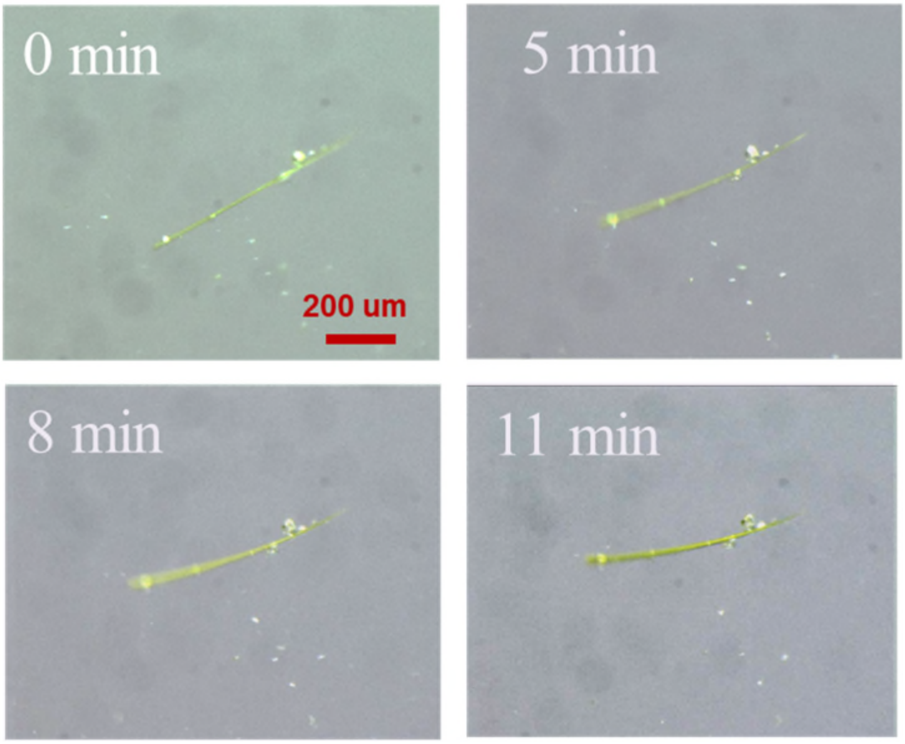

Supplementary Fig. 10 The photoresponsive behavior of U-CB[8]-MPyVB under different UV irradiation time at 25℃ in mineral oil.

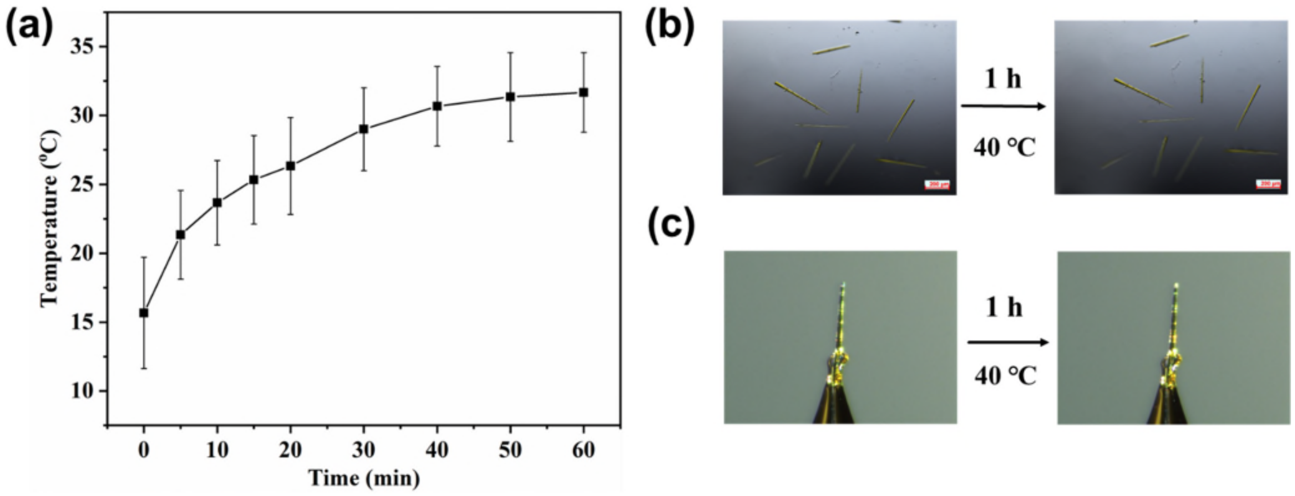

Supplementary Fig. 11 Temperature changes induced by photothermal effect and its possible effects on the motion of the crystal under UV lamp (365 nm, power of 6 W) irradiation condition. **(a)** The temperature changes at different irradiation time induced by photothermal effect (each error bar represents the standard deviation of temperature at a certain time point). Error bars represent the mean ± S.D. of three independent experiments. **(b-c)** Changes of crystals immersed in mineral oil **(b)** and fixed on the Loop **(c)** when subject to heated atmosphere at 40 ℃ after 60 minutes.

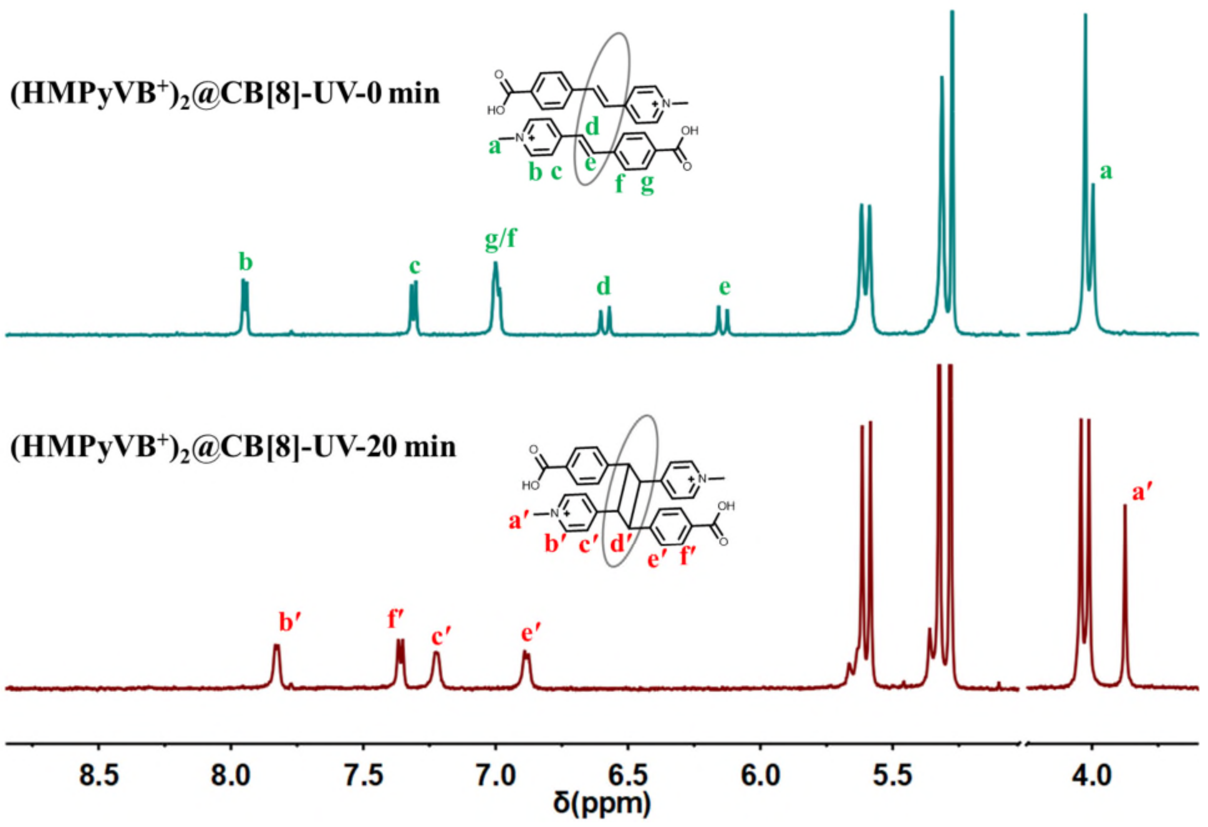

Supplementary Fig. 12 ^{1}H NMR spectrum of model complex $(HMPyVB^{+})_2$@CB[8] in solution before and after UV radiation at 25 °C (D_2O, 500 M Hz).

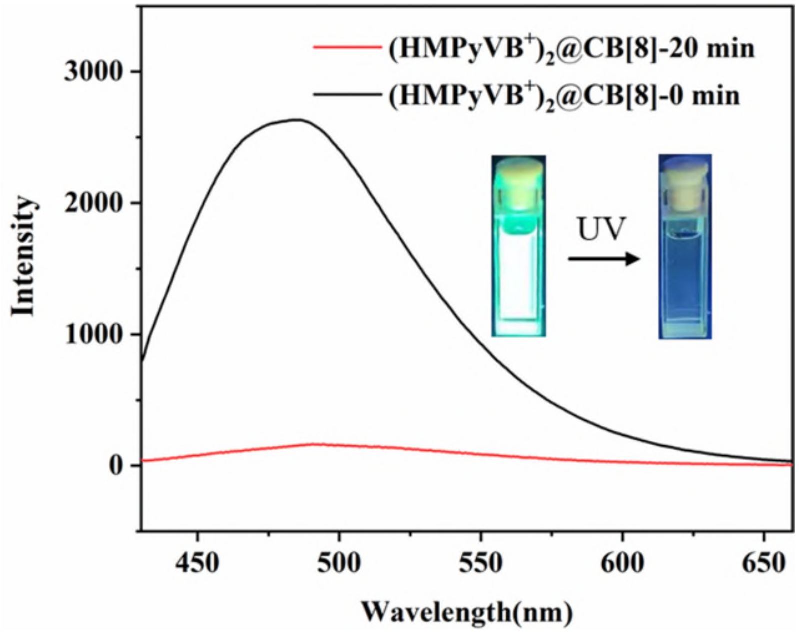

Supplementary Fig. 13 Fluorescence spectra of model complex $(HMPyVB^+)_2$@CB[8] in solution before and after UV radiation.

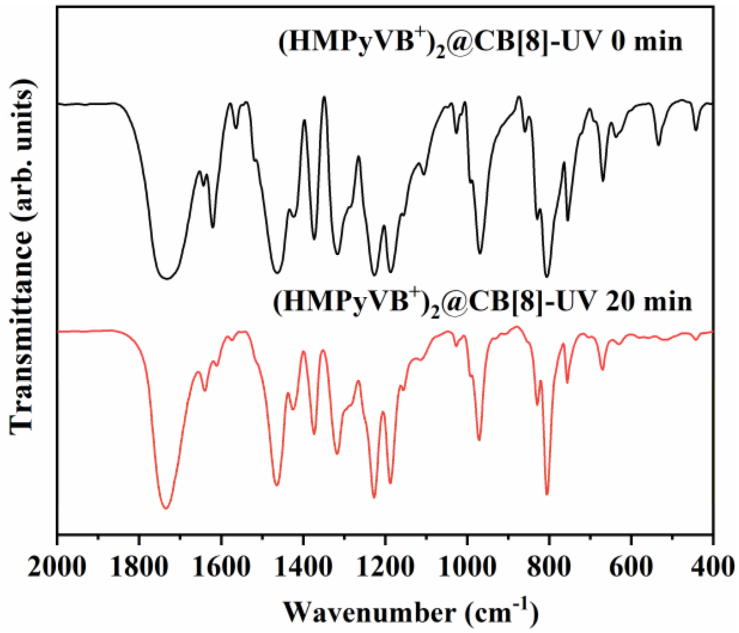

Supplementary Fig. 14 FT-IR spectrum of model complex $(HMPyVB^+)_2$@CB[8] in solution before and after UV radiation.

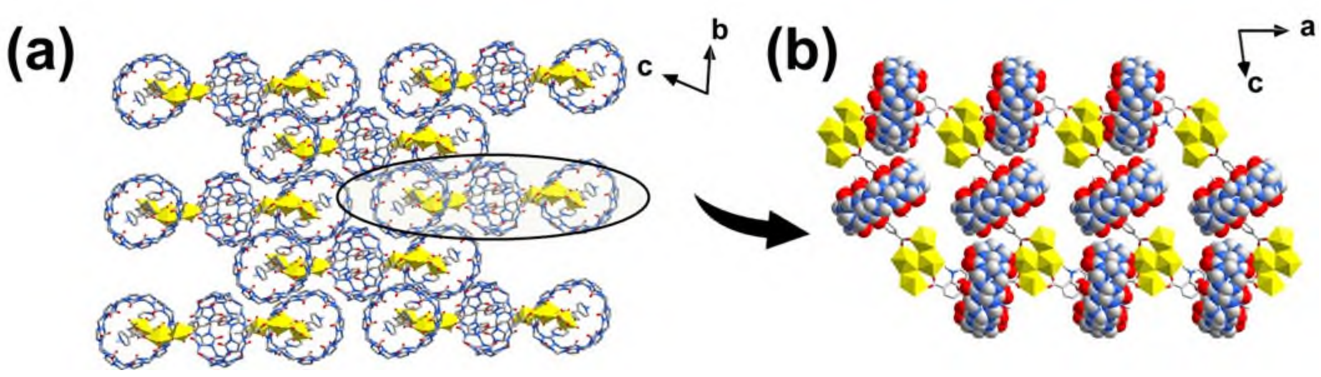

Supplementary Fig. 15 Single crystal structure of U-CB[8]-MPyVB-A. (a) The packing mode of U-CB[8]-MPyVB-A. (b) One-dimensional chain of U-CB[8]-MPyVB-A connected by supramolecular and coordination interaction. (Oxygen atom: red; Carbon atom: gray; Nitrogen atom: blue; uranium atom: yellow).

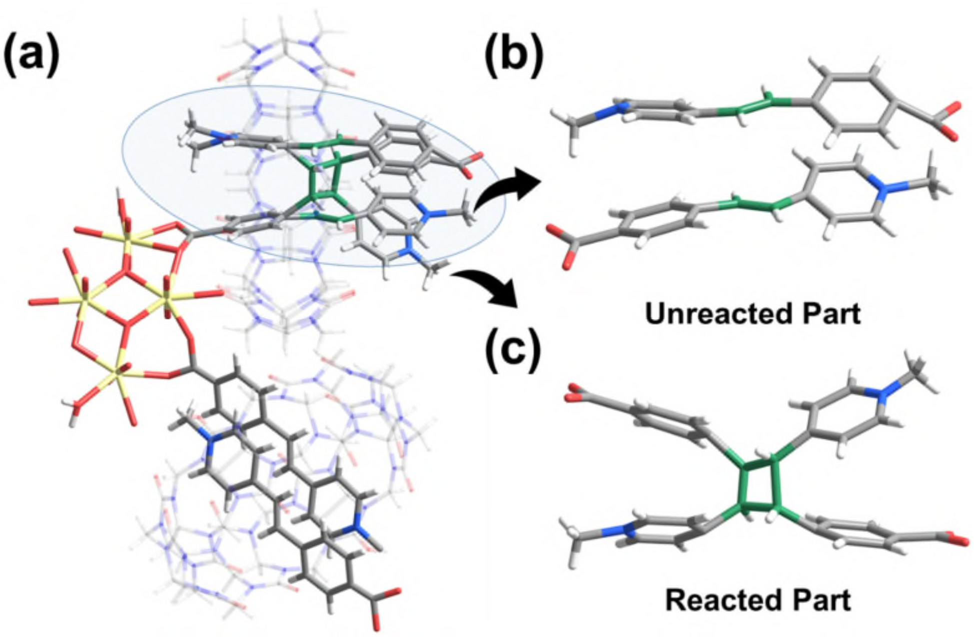

Supplementary Fig. 16 Single crystal structure of U-CB[8]-MPyVB-Int. (a) Asymmetric unit of U-CB[8]-MPyVB-Int. Crystal structure of photoactive motif of U-CB[8]-MPyVB-Int containing two possible different parts, unreacted part and reacted part. **(b-c)** Two possible parts of photoactive motif in U-CB[8]-MPyVB-Int: unreacted part (b) and dimerized part (c) (oxygen atom: red; carbon atom: gray; nitrogen atom: blue; uranium atom: yellow).

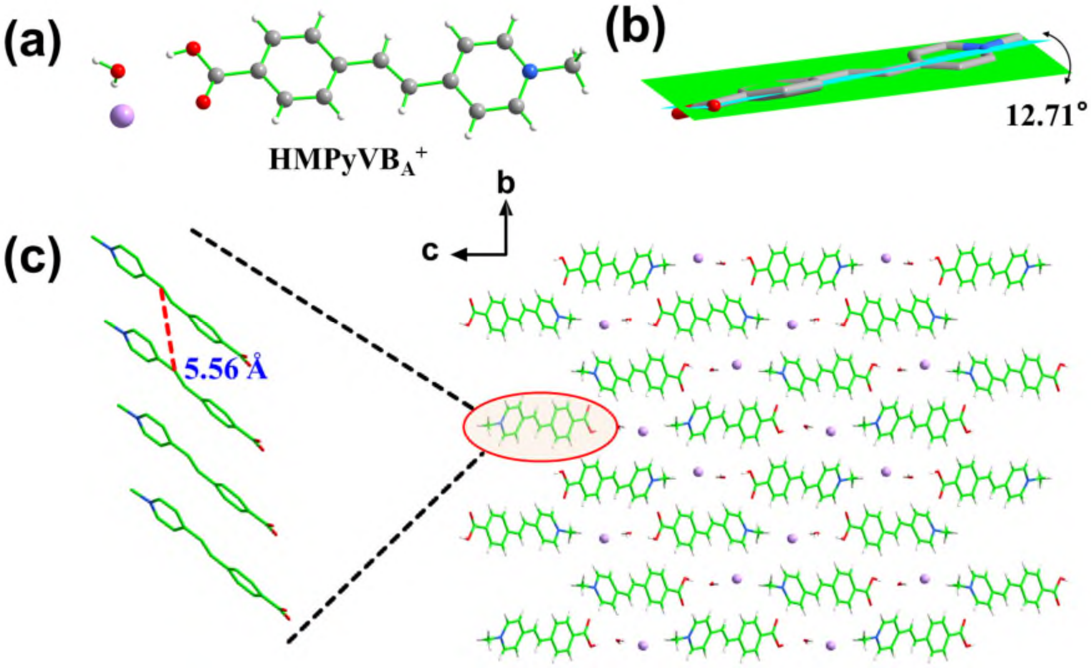

Supplementary Fig. 17 Single crystal structure diagram of [HMPyVB]I. (a) Asymmetric unit. (b) The dihedral angle between two benzene rings in [HMPyVB]I. (c) The packing mode of [HMPyVB]I crystal (The enlarged area represents the distance between C=C between two $HMPyVB^+$ ligands).

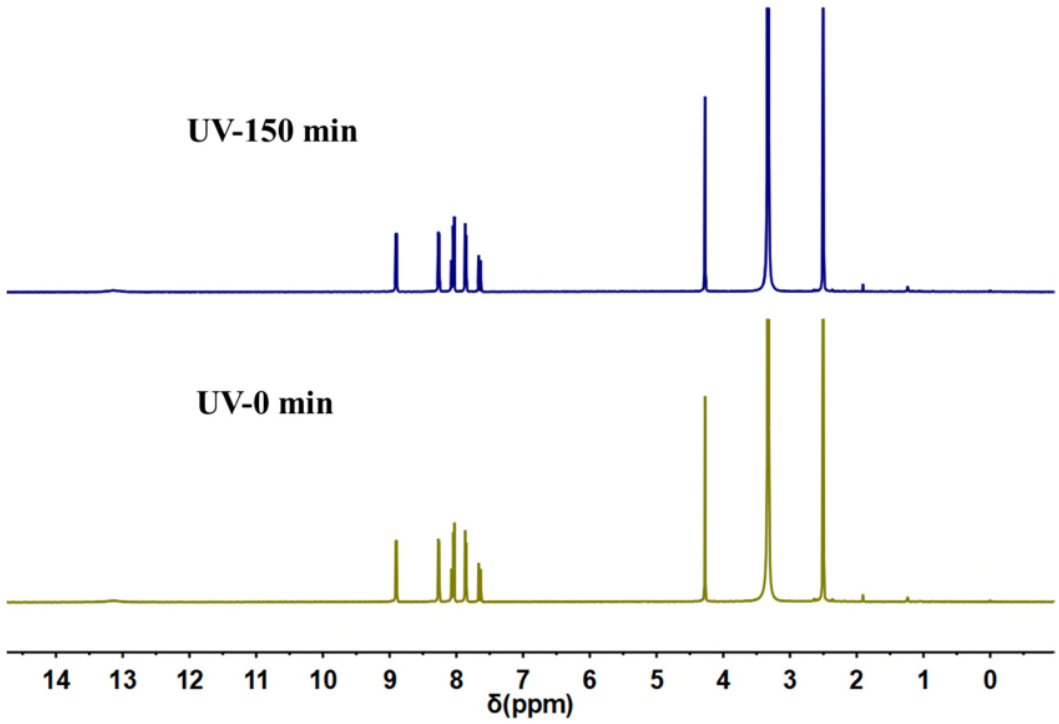

Supplementary Fig. 18 ^{1}H NMR spectra of crystalline [HMPyVB]I before and after UV radiation at 25 °C (DMSO-d_6, 500 M Hz, crystalline [HMPyVB]I was dissolved by DMSO-d_6 before and after UV radiation).

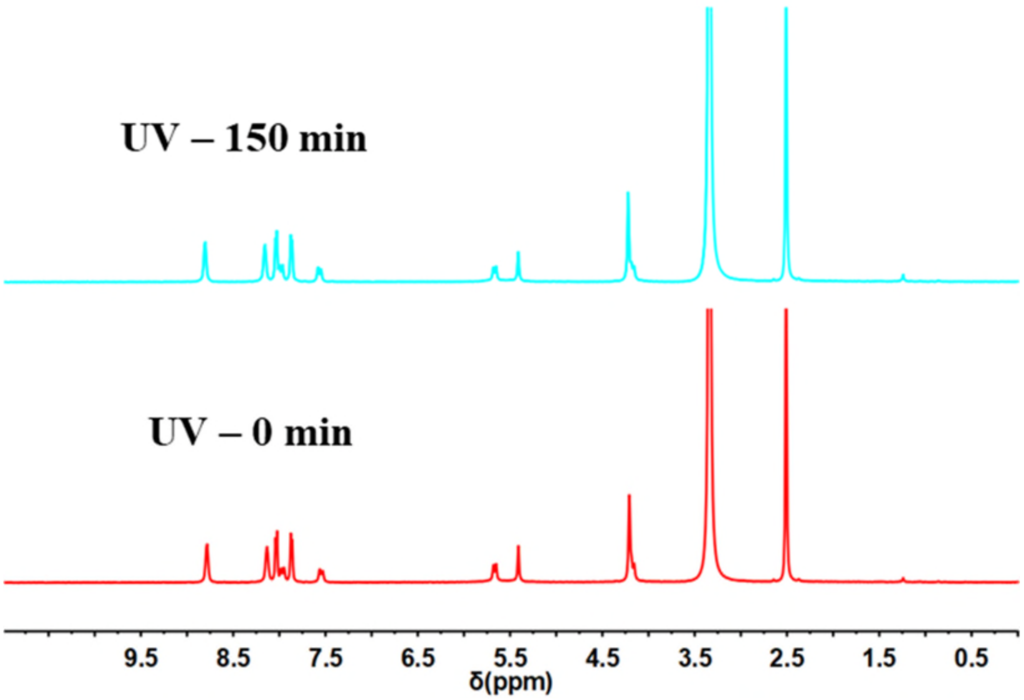

Supplementary Fig. 19 ^{1}H NMR spectra of crystalline CB[8]-HMPyVB before and after UV radiation at 25 °C (DMSO-d_6, 500 M Hz, CB[8]-HMPyVB was dissolved by DMSO-d_6 before and after UV radiation).

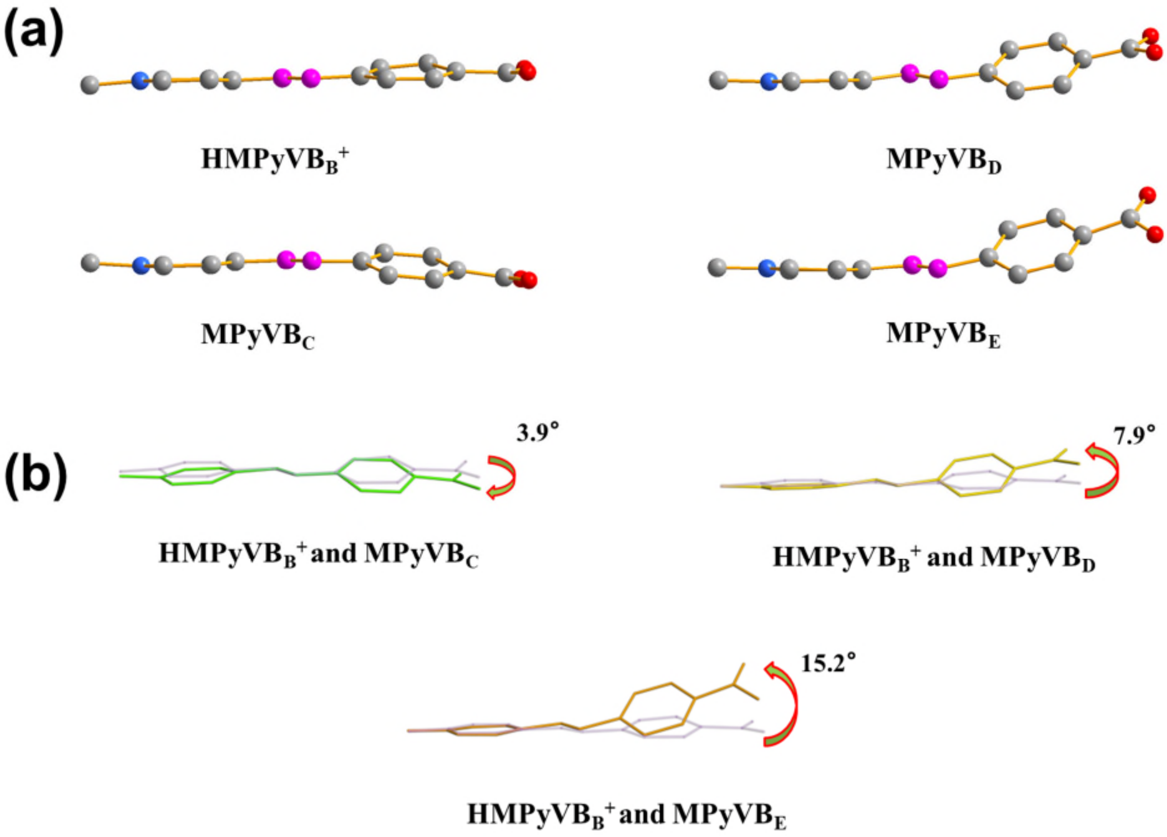

Supplementary Fig. 20 $HMPyVB^+$ or MPyVB motifs with different conformations in different environments. **(a)** Ball and stick model of $HMPyVB_B^+$, $MPyVB_C$, $MPyVB_D$ and $MPyVB_E$. **(b)** The rotation angle diagram of $MPyVB_{C,\ D,\ E}$ relative to $HMPyVB_B^+$ after participating in the coordination. ($HMPyVB_B^+$, purple; $MPyVB_C$, green; $MPyVB_D$, yellow; $MPyVB_E$, orange)

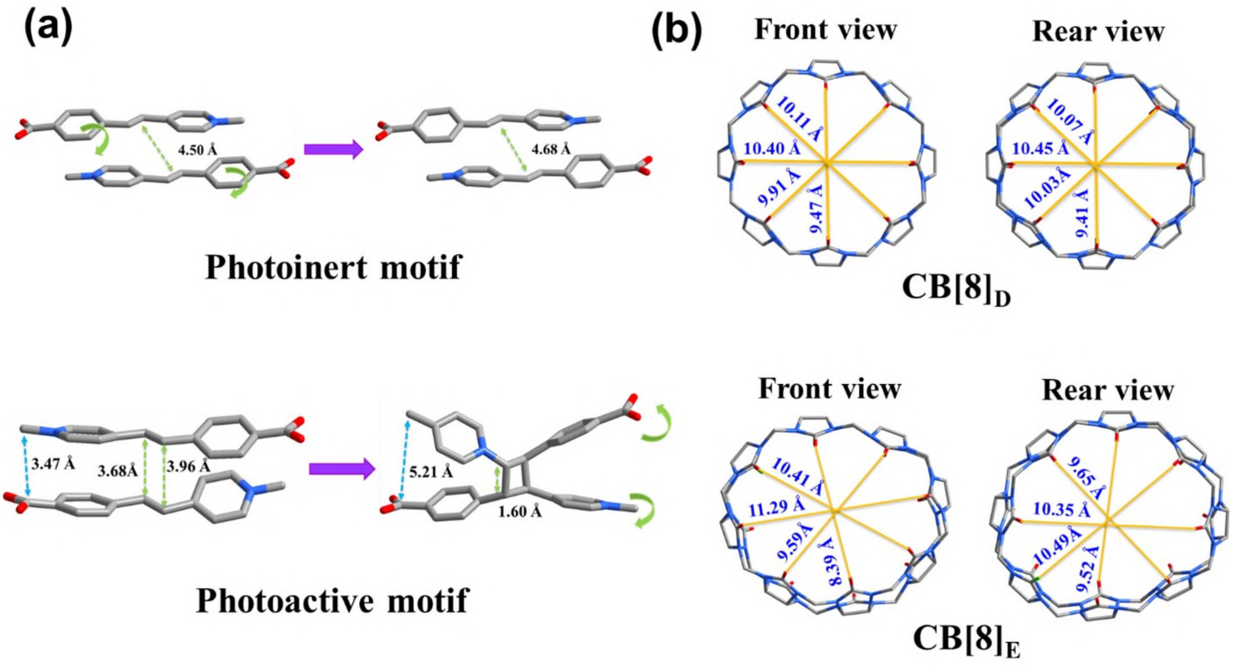

Supplementary Fig. 21 The changes of photoinert motif and photoactive motif before and after UV irradiation. **(a)** Changes of MPyVB in photoinert and photoactive motif after UV irradiation. **(b)** Front and rear views of adaptive changes made by CB[8] in U-CB[8]-MPyVB-A. (CB[8]$_D$, from photoinert motif of U-CB[8]-MPyVB-A; CB[8]$_E$, from photoactive motif of U-CB[8]-MPyVB-A.)

Supplementary Tables

Supplementary Table 1 Distances between C=C bonds and dihedral angles between two benzene rings of $HMPyVB^+$ or MPyVB ligands in diffirent enviroments.

Compound	[HMPyVB]I	CB[8]-HMPyVB	U-CB[8]-MPyVB		U-CB[8]-MPyVB-A	
Functional motif	$HMPyVB_A^+$	$HMPyVB_B^+$	$MPyVB_C$	$MPyVB_{D, E}$	$MPyVB_F$	bisMPyVB
D(C…C)（Å）	5.56	4.44	4.50	3.69, 3.96	4.68	1.58-1.60
Angel (°)	12.71	8.77	17.48	31.27, 36.42	24.03	-

Supplementary Table 2 Distances between O...O in front and back views of CB[8] with different conformations (d for front view, d' for back view).

	CB[8]-HMPyVB	U-CB[8]-MPyVB		U-CB[8]-MPyVB-A	
d(O…O)	$CB[8]_A$	$CB[8]_B$	$CB[8]_C$	$CB[8]_D$	$CB[8]_E$
d_1 (Å)	9.35 Å	9.47 Å	8.81 Å	9.41 Å	8.38 Å
d_2 (Å)	10.09 Å	9.91 Å	10.21 Å	10.03 Å	9.59Å
d_3 (Å)	10.49 Å	10.40 Å	10.90 Å	10.45Å	11.29 Å
d_4 (Å)	10.10 Å	10.11 Å	9.76 Å	10.07 Å	10.41 Å
d_1' (Å)	9.35 Å	9.47 Å	9.10 Å	9.41 Å	9.52 Å
d_2' (Å)	10.09 Å	10.11 Å	10.10 Å	10.03 Å	9.65Å
d_3' (Å)	10.49 Å	10.40 Å	10.41 Å	10.45Å	10.35 Å
d_4' (Å)	10.10 Å	9.91 Å	9.94 Å	10.07 Å	10.49 Å
d_{max}/d_{min}	1.12	1.10	1.24	1.11	1.35
d'_{max}/d'_{min}	1.12	1.10	1.14	1.11	1.10

Supplementary Table 3 Crystal and refinement data of [HMPyVB]I, CB[8]-HMPyVB, U-CB[8]-MPyVB, U-CB[8]-MPyVB-Int and U-CB[8]-MPyVB-A.

	HMPyVB	CB[8]-HMPyVB	U-CB[8]-MPyVB	U-CB[8]-MPyVB-Int	U-CB[8]-MPyVB-A
Formula	$C_{15}H_{16}INO_3$	$C_{39}H_{38}I_{0.5}N_{17}O_{10}$	$C_{117}H_{119}N_{51}O_{45}U_4$	$C_{117}H_{119}N_{51}O_{45}U_4$	$C_{117}H_{119}I_{0.5}N_{51}O_{45}U_4$
fw	385.19	968.31	3911.75	3907.71	3975.19
crystal sys	monoclinic	orthorhombic	triclinic	triclinic	triclinic
space group	$P2_1/c$	Pccn	P-1	P-1	P-1
a, Å	5.5568(3)	26.301(14)	18.5526(13)	18.682(2)	18.7156(14)
b, Å	17.9181(10)	26.420(12)	21.2051(13)	20.997(3)	20.6650(13)
c, Å	15.7506(7)	13.664(7)	24.2208(17)	24.262(3)	24.2449(17)
α, degree	90	90	70.056(3)	69.945(4)	70.156(2)
β, degree	90.584(2)	90	80.150(3)	80.142(4)	80.432(3)
γ, degree	90	90	85.702(2)	84.323(5)	83.129(3)
V, Å^3	1568.16(14)	9495(8)	8823.8(10)	8799.9(18)	8677.2(11)
T, K	273.15	295.28	170.0	170.0	170.0
F (000)	760.0	3980.0	3812.0	3804.0	3865.0
Dc (g/cm^3)	1.632	1.355	1.472	1.475	1.521
μ (mm^{-1})	2.048	0.421	3.742	3.752	3.894
GooF (S)	1.263	1.158	1.018	1.068	1.028
Rint	0.0568	0.0732	0.0669	0.0936	0.0891
*R*1, *wR*2 (I>=2σ(I))	0.0457, 0.0557	0.0771, 0.1740	0.0332, 0.0709	0.0926, 0.2192	0.0480, 0.1100
*R*1, *wR*2 (all data)	0.0619, 0.0588	0.0879, 0.1805	0.0481, 0.0794	0.1218, 0.2304	0.0666, 0.1214

Supplementary Table 4 Comparison of cell parameters and response time between U-CB[8]-MPyVB and other photobending materials before and after photodimerization.

	U-CB[8]-MPyVB			Zn(bdc)(3F-spy)			BOACl24		
Parameter	Before	After	Rate	Before	After	rate	Before	After	rate
V, Å^3	8823.8	8677.2	-1.66	3908.3	3669.7	-6.10	644.6	1345.2	+108.69
a, Å	18.55	18.72	+0.92	21.56	20.12	-6.70	3.87	9.83	+154.01
b, Å	21.21	20.67	-2.55	10.98	10.88	-0.91	11.71	12.17	+3.93
c, Å	24.22	24.25	+0.12	18.73	18.87	0.75	15.22	13.42	-11.83
Time	1380 s			13 s			40 s		
Parameter	BTAF24			BTAF4			E-BDHF		
V, Å^3	621.2	1184.6	+90.70	1226.2	1876.1	+53.00	874.6	841.2	−3.82
a, Å	6.58	12.79	+94.38	6.14	10.02	+63.19	10.938	11.769	+7.59
b, Å	7.54	11.15	+47.88	14.56	13.69	-5.98	5.933	6.0891	+2.63
c, Å	12.69	8.42	-33.65	13.79	15.40	-7.98	14.230	12.403	−12.84
Time	14 s			31 s			64 s		

Supplementary references

1. Yin H, *et al.* Chameleonic Dye Adapts to Various Environments Shining on Macrocycles or Peptide and Polysaccharide Aggregates. *ACS. Appl. Mater. Interfaces.* **9,** 33220-33228 (2017).
