# Supplementary material for: Controllable photomechanical bending of metal-organic rotaxane crystals facilitated by regioselective confined-space photodimerization
Source: Nat Commun. 2022 Apr 19;13:2030. doi: 10.1038/s41467-022-29738-y (PMC9019062; doi:10.1038/s41467-022-29738-y)
Supplement: Supplementary file 3 — Description of Additional Supplementary Files [file 41467_2022_29738_MOESM3_ESM.pdf]

Description of Additional Supplementary Files

Supplementary Movie 1. The photoresponsive behavior of a U-CB[8]-MPyVB crystal in mineral oil. A rod-shaped crystal of U-CB[8]-MPyVB immersed in mineral oil and irradiated by UV light (365 nm, 6 W). The crystal moved around in the mineral oil, accompanied by slight macroscopic bending within 11 minutes (The movie is played at 64x speed).

Supplementary Movie 2. The photoresponsive behavior of crystal-A in air. One end of Crystal-A with a length of about 320 μm was fixed on the test platform in air so that it stands vertically and then subject to incident light in specific direction (365 nm, 6 W). Crystal-A bends slowly from a straight line towards the direction of incident light when exposed to ultraviolet light within 10 minutes. (The movie is played at 64x speed).

Supplementary Movie 3. The photoresponsive behavior of crystal-B in air. One end of Crystal-B with a length of about 720 μm was fixed on the test platform in air so that it stands vertically and then subject to incident light in specific direction (365 nm, 6 W). Crystal-B bends slowly from a straight line towards the direction of incident light when exposed to ultraviolet light within 20 minutes. (The movie is played at 64x speed).

Supplementary Movie 4. The photoresponsive behavior of crystal-C in air. One end of Crystal-C with thicker trunk was fixed on the test platform in air so that it stands vertically and then subject to incident light in specific direction (365 nm, 6 W). Crystal-C bends slowly from a straight line towards the direction of incident light when exposed to ultraviolet light within 23 minutes. (The movie is played at 64x speed).
